# Rapid generation of drug-resistance alleles at endogenous loci using CRISPR-Cas9 indel mutagenesis

**DOI:** 10.1371/journal.pone.0172177

**Published:** 2017-02-23

**Authors:** Jonathan J. Ipsaro, Chen Shen, Eri Arai, Yali Xu, Justin B. Kinney, Leemor Joshua-Tor, Christopher R. Vakoc, Junwei Shi

**Affiliations:** 1 Cold Spring Harbor Laboratory, Cold Spring Harbor, New York, United States of America; 2 Department of Cancer Biology, Abramson Family Cancer Research Institute, Perelman School of Medicine, University of Pennsylvania, Philadelphia, Pennsylvania, United States of America; 3 Molecular and Cellular Biology Program, Stony Brook University, Stony Brook, New York, United States of America; 4 W. M. Keck Structural Biology Laboratory, Cold Spring Harbor Laboratory, Howard Hughes Medical Institute, Cold Spring Harbor, New York, United States of America; SRI International, UNITED STATES

## Abstract

Genetic alterations conferring resistance to the effects of chemical inhibitors are valuable tools for validating on-target effects in cells. Unfortunately, for many therapeutic targets such alleles are not available. To address this issue, we evaluated whether CRISPR-Cas9-mediated insertion/deletion (indel) mutagenesis can produce drug-resistance alleles at endogenous loci. This method takes advantage of the heterogeneous in-frame alleles produced following Cas9-mediated DNA cleavage, which we show can generate rare alleles that confer resistance to the growth-arrest caused by chemical inhibitors. We used this approach to identify novel resistance alleles of two lysine methyltransferases, DOT1L and EZH2, which are each essential for the growth of MLL-fusion leukemia cells. We biochemically characterized the DOT1L mutation, showing that it is significantly more active than the wild-type enzyme. These findings validate the on-target anti-leukemia activities of existing DOT1L and EZH2 inhibitors and reveal a simple method for deriving drug-resistance alleles for novel targets, which may have utility during early stages of drug development.

## Introduction

One key aspect of evaluating small molecule inhibitors is the verification of on-target effects in cells. This can be addressed by generating genetic alterations conferring resistance to the inhibitors, and testing whether expression of such alleles can rescue the cellular effects of the inhibitor. Currently, there are two conventional strategies for generating such chemical inhibitor resistant mutations: (i) Expressing a randomly mutagenized cDNA library of the suspected drug target in cells, followed by selection for resistance phenotypes [1,2], or (ii) Identifying genetic mutation(s) from spontaneously emerging resistant cell populations produced under continuous exposure to the chemical inhibitor [3]. While both methods have been successfully employed in identifying chemical inhibitor resistant mutations, such as identifying the imatinib-resistant BCR-ABL in chronic myeloid leukemia [1] or the vemurafenib-resistant BRAF mutants in melanoma [4], some limitations still persist. For example, achieving the proper expression level of the ectopically expressed cDNA is critical to confer resistance. Moreover, spontaneous mutations that confer resistance may occur in genes unrelated to the drug’s direct target, as is the case in mutations that induce an up-regulation in transporter proteins that can pump the inhibitor out of the cell.

Here, we present a simple method that employs CRISPR-Cas9 (clustered regularly interspaced short palindromic repeats) mutagenesis to derive resistance-conferring alleles of endogenous genes for demonstrating the on-target effects of compounds in cells. CRISPR-Cas9 is an RNA-guided endonuclease system that is widely used for genome editing [5,6]. In this system, a programmable single guide RNA (sgRNA) directs a Cas9 protein to desired genomic regions and produces a double-strand DNA break (DSB). Through the error-prone non-homologous end joining repair pathway, a collection of indel mutations are introduced to regions flanking the Cas9-mediated DSB site [7–9]. Directing these CRISPR-induced indel mutations to protein-coding regions generates both in-frame and frame-shift mutations of the gene being targeted [10–12]. By taking advantage of functionally intact in-frame indels [11,13], we hypothesized that the diversity of indels induced by CRISPR mutagenesis could be used to select for chemical inhibitor-resistant allele(s), and subsequently, could be used for evaluating the on-target effects of corresponding chemical inhibitors. In this work, we derived reproducible inhibitor-resistant alleles of two lysine methyltransferases (KMT), DOT1L and EZH2, through domain-focused CRISPR indel mutagenesis. Through CRISPR-based positive selection screens, we found a recurring DOT1L mutation, VVEL293MM, which conferred resistance to a DOT1L inhibitor, EPZ-5676 [14]. Biochemical experiments suggested that the DOT1L VVEL293MM mutant was a hypermorphic mutant, as it alleviated the growth arrest effect of EPZ-5676 mainly by increasing the basal level of key methylated DOT1L substrate. Furthermore, we extended our method to identify an EZH2 mutant that rendered leukemia cells resistant to an EZH2 inhibitor, EPZ-6438 [15]. Taken together, we show that domain-focused CRISPR-Cas9 indel mutagenesis allows for a straightforward and rapid identification of drug-resistant alleles, making it a useful tool for the evaluation of on-target drug activity.

## Materials and methods

### Plasmids

The constitutive, human codon-optimized, *Streptococcus pyogenes* Cas9 retroviral expression construct (MSCV-hCas9-PGK-Puro, Addgene: #65655) and lentiviral sgRNA expression vector (LRG, Addgene: #65656) were adapted from previous work [11]. The wild-type human DOT1L cDNA was cloned into a lentiviral expression vector with EFS prompter and P2A-linked Puromycin resistance gene. P2A, porcine teschovirus-1 2A. The wild-type EHZ2 cDNA was cloned into an MSCV-based vector containing a puromycin-resistance gene and a GFP reporter as previously reported [16]. Both the DOT1L VVEL293MM DOT1L and EZH2 TR683KK mutations were introduced by standard PCR mutagenesis. The PCR cloning procedures were performed using the In-Fusion® HD Cloning Kit (Clontech: #638909). For the recombinant DOT1L KMT domain, wild-type and VVEL293MM mutant cDNA were cloned into the bacterial expression vector pET-22b (Novagen) via sequence- and ligation-Independent cloning, which contained an N-terminal His_6_-tag and TEV protease site that allowed for affinity purification and subsequent cleavage of the affinity tag.

### Single and pooled CRISPR sgRNA cloning

Plasmids expressing a single sgRNA were cloned by annealing two DNA oligos and ligating into a BsmB1-digested LRG vector. To improve U6 promoter transcription efficiency, an extra 5’ G nucleotide was added to all sgRNAs that did not start with a 5’ G. For the pooled sgRNAs targeting the KMT domain of either DOT1L or EHZ2, all possible sgRNAs were designed based on a PAM sequence of NGG that is recognized by the *S*. *pyogenes* Cas9 protein. The sgRNAs were synthesized individually, annealed to complementary sgRNAs, pooled in equal molar ratios, and ligated into BsmB1-digested LRG vectors. All sgRNA sequences used in this study are provided in S1 Table.

### Cell culture and virus production

All cell lines used in this study were tested as mycoplasma negative. The RN2c cell line was adapted from previous study [11]. Briefly, murine MLL-AF9/Nras^G12D^ acute myeloid leukemia cells (RN2) [17] were transduced with MSCV-hCas9-PGK-Puro, followed by puromycin selection. A single cell-derived clone was derived by serial dilution. RN2 and RN2c cells were cultured in RPMI1640 supplemented with 10% fetal bovine serum (FBS) and penicillin/streptomycin.

Plat-E cells were used for retroviral delivery of hCas9 or EZH2 cDNA expression vectors, following standard procedures [18]. HEK293T cells were used for lentiviral production of sgRNA or DOT1L expression vectors. Ecotropic Plat-E cells and HEK293T cells were cultured in DMEM supplemented with 10% FBS and penicillin/streptomycin. Lentivirval vectors were mixed with pVSV-G and psPAX2 in a 4:2:3 ratio using PEI reagent (Polysciences, #23966). Viral supernatants were collected at 36, 48, 60, and 72 hours post-transfection. RN2 cells were transduced with empty or indicated cDNA overexpression vectors, followed by puromycin selection for ectopic cDNA overexpression. To measure the relative cell accumulation, cDNA-transduced RN2 cells were counted at the initial and end experimental time points using a Guava Easycyte HT instrument (Millipore). Gating was performed on live cells using forward and side scatter together with propidium iodide to exclude dead cells. The relative cell accumulation of each experimental condition was calculated by normalizing to the untreated condition.

### Pooled sgRNA screening, Miseq library construction, and data analysis

Virus containing the pooled sgRNA library was generated as described above. Serial dilution of this virus in correlation with the GFP+ cell population was used to estimate the viral titer multiplicity of infection (MOI). In the initial infected cell population, the total number of RN2c cells corresponded to an approximately 1 million-fold representation of each sgRNA. To ensure that a single sgRNA was transduced per cell, the viral volume for infection corresponded to an MOI of 0.5. At 3 days post-infection, RN2c cells were treated with chemical inhibitors, while a small portion of the RN2c cells was harvested and used as a reference time point (day 0). Likewise, at later time points, when the sgRNA+/GFP+ and inhibitor-resistant cell population emerged, a small portion of the cells was harvested again and saved for subsequent deep sequencing experiments. The sgRNA+/GFP+ cell population was monitored over the experimental time course using a Guava Easycyte HT instrument (Millipore). Gating was performed on live cells using forward and side scatter together with propidium iodide to exclude dead cells, prior to measurement.

The sgRNA cassette MiSeq deep sequencing library was constructed using a similar method as previously described [11]. Genomic DNA was extracted using QiAamp DNA mini kit (Qiagen #51304), following the manufacturer’s protocol. In order to maintain >1000-fold sgRNA library representation, 20 parallel PCR reactions were performed to amplify the sgRNA cassette using the 2X High Fidelity Phusion Master Mix (Thermo Scientific #F-548). PCR products were subjected to Illumina MiSeq library construction and sequencing. First, the PCR product was end repaired with T4 DNA polymerase (New England BioLabs, NEB), DNA polymerase I (NEB), and T4 polynucleotide kinase (NEB). Then, an adenosine overhang was added to the end-repaired DNA using Klenow DNA Pol Exo- (NEB). The overhanging DNA fragment was ligated with diversity-increased barcoded Illumina adaptors followed by seven pre-capture PCR cycles. The barcoded libraries were pooled at an equal molar ratio and subjected to massively parallel sequencing through a MiSeq instrument (Illumina) using paired-end 75-bp sequencing (MiSeq Reagent Kit v3; Illumina MS-102-3001).

The sequence data were de-barcoded and trimmed to contain only the sgRNA sequence, and subsequently mapped to the reference sgRNA library without allowing any mismatches. The read counts were calculated for each individual sgRNA. To calculate the relative abundance of each sgRNA, the read counts of each sgRNA were divided by the total read counts of the pooled sgRNA library. The primer and diversity-increased barcode information are listed in S1 Table.

### MiSeq analysis of CRISPR-induced mutations

A deep sequencing method was used to identify the DOT1L mutants associated with EPZ-5676 resistance, as described previously [11]. The genomic region of *DOT1L* exon 8 was PCR amplified with High Fidelity 2X Phusion Master Mix (Thermo Scientific #F-548) and subjected to MiSeq library construction as described above. Barcoded libraries were pooled at an equimolar ratio and subjected to massively parallel sequencing by MiSeq (Illumina) using paired-end 150-bp sequencing (MiSeq Reagent Kit v2; Illumina MS-102-2002).

Data analysis was performed as described previously [11]. Paired-end Illumina reads were aligned to the DOT1L genomic locus and variant sequences were called. Variant sequences were recorded in the tab “DOT1L mutations pooled screen” of (for the pooled sgRNA experiment) or in tab “DOT1L mutations sgRNA#47” of (for the single sgRNA experiment) S1 Table,), if they were present in at least a total of 10 reads across the two time points and were not present in the control sample.

### Real-time PCR

RNA was prepared using Trizol reagent (Invitrogen). cDNA synthesis was performed using qScript cDNA SuperMix (Quanta Biosciences, #101414–106). Quantitative PCR (qPCR) analysis was performed on an ABI 7900HT with SYBE green (ThermoFisher, #4309155). All signals were quantified using the delta-Ct method and normalized to the levels of Gapdh.

### Expression and purification of recombinant DOT1L KMT domain

The DOT1L KMT domain constructs were transformed into *E*. *coli* BL21 (DE3)-RIPL cells for protein expression. Cultures were grown in TB media with antibiotic at 37°C until the OD_600_ was approximately 1.5. The cultures were then briefly cooled on ice and treated with 0.5 mM isopropyl-β-d-1-thiogalactopyranoside (IPTG) to induce protein expression. Cultures were then incubated overnight at 16°C with shaking. After expression, the cell pellets were thawed, resuspended in 20 mM Tris, pH 8.0, 500 mM NaCl, 10 mM imidazole, 10% glycerol, 5 mM βME (~20 mL per liter culture), and lysed by sonication. The cell lysate was clarified by ultracentrifugation at 140,000 g for 30 min and the supernatant was applied to a Ni-NTA (Qiagen) column equilibrated with lysis buffer. The bound proteins were washed with lysis buffer, further washed with lysis buffer containing 30 mM imidazole, and finally eluted in lysis buffer containing 200 mM imidazole. The His_6_ tag was then removed by the addition of TEV protease (1:20 m/m ratio protease:DOT1L KMT) and overnight incubation at 4°C.

The proteins were further purified by ion exchange chromatography using a MonoS 5/50 GL column (GE Healthcare) equilibrated with 20 mM HEPES, pH 7.5, 1 mM EDTA, 1 mM DTT. Bound proteins were fractionated by elution using a gradient of NaCl from 0 to 1 M. Fractions corresponding to the target protein (around 45 mS) were pooled, concentrated, and further purified by gel filtration using a Superdex200 increase column (GE Healthcare) equilibrated with 20 mM HEPES, pH 7.5, 200 mM NaCl, 1 mM EDTA, 1 mM DTT.

The purified protein concentrations were determined by A_λ = 280_ measurement and corrected for the calculated molecular extinction coefficient. The proteins were then concentrated to 10–20 mg/mL and stored at 4°C until needed. Typical yields (with >98% purity as assessed by SDS–PAGE) were 4 mg of the wild-type protein and 0.5 mg of the VVEL293MM mutant protein per liter culture.

### Methyltransferase activity assay

Methyltransferase activity assays with the purified DOT1L KMT domains were performed on native nucleosome substrates purified from human HeLa cells (BPS Bioscience) using tritiated S-adenosyl methionine (^3^H-SAM) (Perkin Elmer). To accurately determine the relative methyltransferase activities of the wild-type and VVEL293MM mutant proteins, serial dilutions of the enzymes were made under the same conditions. Each 10 μL reaction included 2 μL of ^3^H-SAM (1 μCi, ~1.3 mM final concentration) and 1 μg of the nucleosome substrate in a buffer of 20 mM Tris, pH 8.0, 4 mM EDTA, 1 mM DTT, 0.01% Triton X-100. The enzyme concentration varied as a two-fold dilution series from 100 nM to 6.25 nM. After mixing all components, the reactions were incubated at room temperature for 2 hours. Each reaction was then denatured and subjected to SDS-PAGE followed by gel fixation, treatment with ENLIGHTNING autoradiography enhancer (Perkin Elmer), drying, and autoradiography. The dried gels were then exposed to BioMax XAR Film (Kodak, Carestream) for approximately 60 hours. After developing, the films were scanned and band intensities were quantified using the ImageQuant TL 8.1 analysis software package (GE Healthcare). The presented data reflect the average of two replicates, error bars indicate the standard error of the mean, and the linear fit of the data was constrained to pass through the origin.

Subsequently, to establish if the wild-type and mutant proteins exhibited differential sensitivity to inhibitors, similar reactions were carried out in the presence of inhibitors at a fixed protein concentration of 50 nM. As the relative activity of the VVEL293MM enzyme was found to be roughly 3-fold higher than that of the wild-type protein, we sought to normalize the total activity observed on our autoradiographs to better discern slight differences in the proteins’ sensitivity to inhibitors. To accomplish this, reactions volumes were scaled such that three-fold more material was loaded per gel lane for the wild-type samples. Inhibitor concentrations ranged from 400 nM to 50 nM as a two-fold dilution series. Sample processing was performed as described above. The integrated band intensity for the uninhibited reactions was then used to normalize the intensities of the inhibitor concentration series.

### Probing neomorphic methyltransferase activity with mass spectrometry

#### Reaction preparation

To determine if the mutant enzyme exhibits a neomorphic activity compared to the wild-type enzyme, a proteomic analysis of the methylation reactions was performed. The nucleosome substrate (10 μg) in a buffer of 20 mM Tris, pH 8.0, 4 mM EDTA, 1 mM DTT, 0.01% Triton X-100 was reacted with the DOT1L wild-type enzyme (10 μM final concentration) in the presence of (non-radiolabeled) S-adenosyl methionine at a concentration of 1 mM for two hours at room temperature in a reaction volume of 20 μL.

#### Precipitation, digestion and iTRAQ labeling

Tris(2-carboxyethyl)phosphine (TCEP) was added to each reaction at a final concentration of 5 mM. The samples were heated at 55°C for 20 min then allowed to cool to room temperature. Methyl methanethiosulfonate (MMTS) was next added to a final concentration of 10 mM and the samples were incubated at room temperature for 20 min to block free sulfhydryl groups. Proteins were then precipitated by the addition of 10 volumes of 10% trichloroacetic acid (TCA) in acetone followed by incubation at -20°C for overnight. The samples were then centrifuged at 14,000 for 10 min at 4°C and the supernatant were removed. Ten volumes of ice cold acetone were added to the pellet which was then vortexed. The precipitate was incubated at -20°C for 10 minutes before centrifuging at 4°C and 14,000g for 5 minutes. The supernatant was removed and discarded without disturbing the pellet, and the pellet was allowed to dry. The precipitated proteins were resuspended with 50 μL of 100 mM TEAB. The pellet was then sonicated until completely dissolved. 2 μg of sequencing grade trypsin (Promega) was then added to the samples. Digestion continued at 37°C overnight after which the samples were and dried in vacuo. Peptides were resuspended in 50 μL of 0.5M TEAB/70% ethanol and labeled with 8-plex iTRAQ reagents for 2 hours at room temperature essentially according to Ross et al (2004). Labeled samples were then acidified to pH 4 using formic acid, combined, and concentrated in vacuo until ~10 μL remained.

#### Mass spectrometry

A Q-Exactive High Field mass spectrometer (Thermo Scientific) equipped with a nano-ion spray source was coupled to an EASY-nLC 1200 system (Thermo Scientific). The nano-flow LC system was configured with a self-pack PicoFrit™ 75 μm analytical column with an 8 μm emitter (New Objective, Woburn, MA) packed to 25 cm with ReproSil-Pur C18-AQ, 1.9 μM material (Dr. Maish GmbH). Mobile phase A consisted of 2% acetonitrile; 0.1% formic acid and mobile phase B consisted of 90% acetonitrile; 0.1% formic acid. Peptides were then separated using the following linear gradient steps at a flow rate of 200 nL/min: 2% B to 6% B over 1 min, 6% B to 30% B over 84 min, 30% B to 60% B over 9 min, 60% B to 90% B over 1 min, held at 90% B for 5 min, 90% B to 50% B over 1 min and then held at 50% B for 9 min.

Eluted peptides were directly electrosprayed into the Q-Exactive High Field mass spectrometer with the application of a distal 2.3 kV spray voltage and a capillary temperature of 300°C. Each full-scan mass spectrum (Res = 60,000; 380–1700 m/z) was followed by MS/MS spectra for the top 20 masses. High-energy collisional dissociation (HCD) was used with the normalized collision energy set to 27 for fragmentation, the isolation width set to 1.2 and activation time of 0.1. A duration of 15 seconds was set for the dynamic exclusion with an exclusion list size of 500, repeat count of 1, and exclusion mass width of 10 ppm. We used monoisotopic precursor selection for charge states 2+ and greater, and all data were acquired in profile mode.

#### Probing neomorphic methyltransferase activity with autoradiography

As an alternative means to determine if the mutant possessed a new activity compared to the wild-type enzyme, nucleosome substrates were first pre-reacted with the wild-type protein. The nucleosome substrate (50 μg) in a buffer of 20 mM Tris, pH 8.0, 4 mM EDTA, 1 mM DTT, 0.01% Triton X-100 was reacted with the DOT1L wild-type enzyme (10 μL final concentration) in the presence of (non-radiolabeled) S-adenosyl methionine at a concentration of 1 mM overnight at room temperature in a reaction volume of 100 μL.

After the overnight pre-reaction, the protein components of the reaction were isolated using 7 kDa molecular weight cutoff Zeba spin desalting columns (ThermoFisher Scientific). The pre-reacted nucleosomes were then used as the substrate for autoradiography assays as described above.

## Results

### KMT domain-focused CRISPR indel mutagenesis reveals a DOT1L allele that confers resistance to EPZ-5676-mediated growth arrest

**Fig 1:**
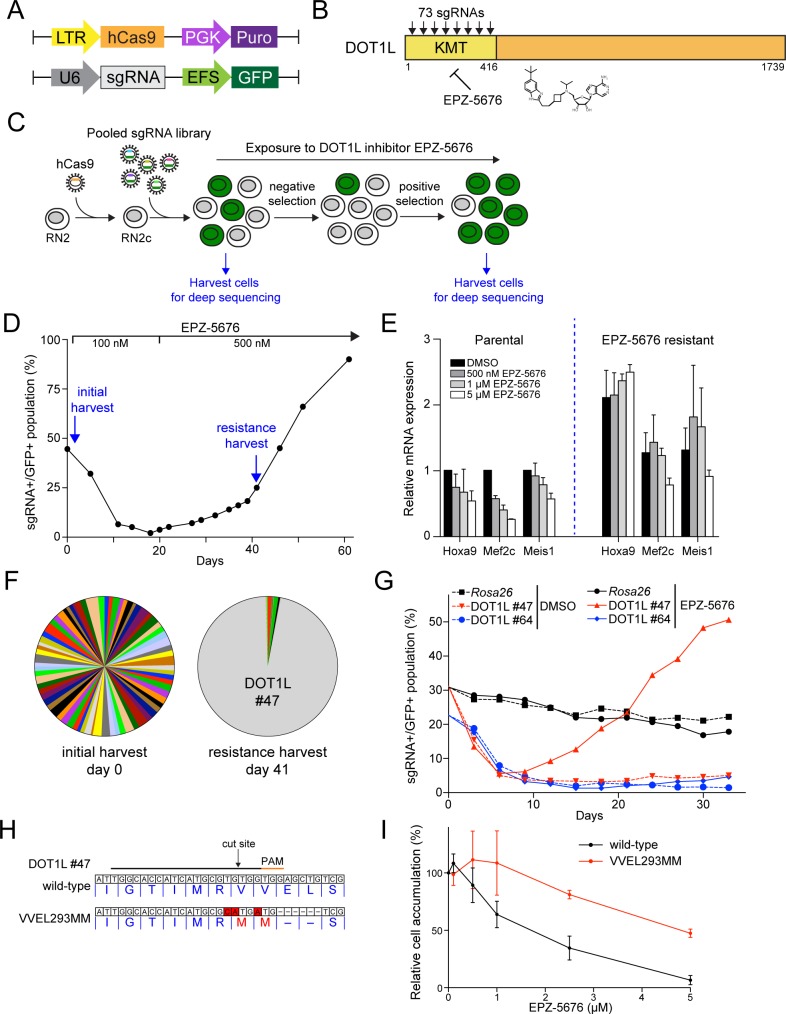
KMT domain-focused CRISPR indel mutagenesis reveals a DOT1L allele that confers resistance to EPZ-5676 mediated growth arrest. (**A**) Schematic of the vector used to derive clonal MLL-AF9; Nras^G12D^ leukemia RN2c cells that express a human codon-optimized *S*. *pyogenes* Cas9 (hCas9) and the vectors used for lentiviral sgRNA transduction. A GFP reporter was used where indicated to track sgRNA positive selection. LTR: long terminal repeat promoter, PGK: phosphoglycerate kinase 1 promoter, Puro: puromycin resistance gene, U6: a Pol III-driven promoter, sgRNA: chimeric single guide RNA, EFS: EF1α promoter, GFP: green fluorescent protein. (**B**) Schematic of DOT1L protein domain. A library of 73 sgRNAs was designed targeting the KMT domain of DOT1L. The DOT1L inhibitor EPZ-5676 targets the KMT domain. KMT, lysine methyltransferase. (**C**) Experimental workflow of domain-focused CRISPR indel mutagenesis screening. A pooled sgRNA library targeting the DOT1L KMT domain was introduced to RN2c via lentiviral transduction, followed by continuous exposure to EPZ-5676. Genomic DNA of indicated cell populations was isolated and harvested for sgRNA cassette quantification and sgRNA-induced indel identification by deep sequencing. (**D**) CRISPR indel mutagenesis genetic screening of DOT1L identified EPZ-5676 resistant mutants. The RN2c cells were treated with EPZ-5676 at day 3 post-transduction, in which a fraction of the cells was harvested and used as a reference time point (day 0). Cells were initially treated with 100 nM of EPZ-5676 at day 0. The inhibitor dosage was increased to 500 nM at day 20. At day 41, an EPZ-5676 resistant GFP+/sgRNA+ population was identified, harvested, and saved for subsequent deep sequencing experiments. (**E**) Quantitative reverse transcription PCR (qRT–PCR) of relative mRNA expression levels in parental and EPZ-5676 resistant RN2c cell lines after a 6-day treatment with indicated the concentration of EPZ-5676 or DMSO. Results were normalized to *Gapdh*, with the relative mRNA level in the DMSO-treated cells set to 1 (*n* = 3). (**F**) Pie charts of the relative abundance for 73 individual sgRNAs at indicated time points. Each slice represents the abundance of a single sgRNA. At day 0 (left), all 73 sgRNAs targeting the DOT1L KMT domain were detected at roughly equal abundance. At day 41 (right), DOT1L sgRNA #47 dominated the population, representing >95% of the GFP-positive cells. (**G**) A proliferation competition assay of RN2c cells transduced with indicated sgRNAs under the treatment of EPZ-5676 or DMSO shows that DOT1L sgRNA #47 induced mutant(s) confer resistance to EPZ-5676. An sgRNA targeting a Rosa locus and a random sgRNA targeting the DOT1L KMT domain (DOT1L sgRNA #64) served as negative controls. (**H**) Nucleotide and protein sequence diagram of the wild-type and DOT1L mutation induced by sgRNA #47 CRISPR mutagenesis under the treatment of EPZ-5676. Indel mutations were observed flanking the sgRNA cut site in exon 8 of DOT1L. The indel mutation corresponded to a VVEL to MM mutation at residue 293. (**I**) The effect of ectopic overexpression of DOT1L wild-type or mutant on RN2c cell proliferation under the treatment of EPZ-5676. RN2c cells were retrovirally transduced with wild-type or mutant human DOT1L cDNA. After a 9-day drug treatment, the relative cell accumulation corresponding to each indicated EPZ-5676 concentrations was measured and normalized to the DMSO treated cells (*n* = 3). All error bars shown represent s.e.m.

To test our strategy for identifying inhibitor-resistant alleles and dissecting the on-target effects of a given chemical inhibitor, we selected the gene encoding DOT1L, a histone H3K79 methyltransferase [19], as our mutagenesis target. The DOT1L protein is essential for the growth of MLL-rearranged leukemia cells and exerts its effect by maintaining active transcription of Hoxa9 and Meis1 [20,21]. There are several selective small molecule inhibitors of DOT1L, including EPZ-5676, which is currently under clinical evaluation for treating MLL- rearranged leukemia (ClinicalTrials.gov identifier NCT02141828). Recent studies have suggested that EPZ-5676 exhibits therapeutic effects in leukemia by inhibiting the methyltransferase activity of DOT1L [14], however this has never formally been proven to be an on-target effect using a resistance-conferring allele of DOT1L. Therefore, we evaluated the CRISPR indel mutagenesis method by targeting DOT1L to generate mutant(s) that confer resistance to EPZ-5676. To begin, we employed a codon optimized *S*. *pyogenes* Cas9-expressing murine MLL-AF9/Nras^G12D^ acute myeloid leukemia cell line (RN2c) [11] (Fig 1A). We designed all possible sgRNAs targeting the methyltransferase domain of DOT1L (73 in total) based on a protospacer adjacent motif (PAM) of NGG that is recognized by the *S*. *pyogenes* Cas9 protein. We cloned these sgRNAs as a pool into a bicistronic lentiviral vector that links each sgRNA to a GFP reporter (Fig 1B). By measuring the GFP-positive cell population, we were able to monitor the abundance of sgRNA positive cells over time in culture. In order to assess a broad spectrum of mutations and potential rare, Cas9-induced, in-frame mutations, we transduced the RN2c cells with the pooled CRISPR lentivirus using approximately 1 million cells per sgRNA. By monitoring the GFP-positive fraction of the cell population, we profiled the abundance of sgRNA positive cells over time (Fig 1C).

We exposed the library-transduced cells to a low concentration of EPZ-5676 (100 nM), which was then stepped-up to a higher concentration of 500 nM at day 20 (Fig 1C and 1D). As expected, we observed that the sgRNA+/GFP+ cells became depleted during initial time points of culturing, consistent with the majority of CRISPR-induced indels within the DOT1L KMT domain resulting in loss-of-function, thereby causing an arrest of growth [11]. After this initial crash of sgRNA+/GFP+ cells, we observed an eventual, progressive, return of sgRNA+/GFP+ cell population at later time points. By day 60 post-transduction, the sgRNA+/GFP+ population had reached >95% (Fig 1D). This pattern of decline and rebound of sgRNA+/GFP+ population was suggestive that rare indel allele(s) of DOT1L produced by Cas9 mutagenesis had conferred resistance to the growth arrest caused by EPZ-5676. Consistent with this possibility, we observed that DOT1L-dependent genes Hoxa9, Meis1, and Mef2c were no longer suppressed by EPZ-5676 in the cells recovered at day 60 of the experiment (Fig 1E).

To gain initial insight into the potential mechanism of CRISPR-Cas9-induced resistance to EPZ-5676, we profiled the representation of the individual sgRNAs over the course of the experiment. To this end, we PCR amplified the sgRNA cassette from genomic DNA that was collected at day 0 and day 41 of the time-course then performed deep sequencing (Fig 1D). While all 73 sgRNAs targeting the DOT1L KMT domain were detected at roughly equal abundance at day 0, we found that a single sgRNA #47 was over-represented in the cell population at day 41 of EPZ-5676 exposure. This suggested the possibility that sgRNA #47 had produced an in-frame indel mutation of DOT1L that conferred resistance to EPZ-5676 (Fig 1F). Since individual sgRNAs tend to produce a reproducible pattern of heterogeneous indel mutations [22], we evaluated whether sgRNA #47 would confer resistance to EPZ-5676 when evaluated individually in an independent experiment compared to control sgRNAs. Indeed, we found that under continuous exposure to EPZ-5676, the leukemia cells transduced with DOT1L KMT-targeting sgRNA #47, but not the control sgRNAs, were initially depleted and later recovered to >50% GFP positivity by day 30 in culture (Fig 1G and S1 Fig). This result suggests that sgRNA #47 generates largely loss-of-function alleles of DOT1L, but at a reproducibly low frequency produces an allele of DOT1L that confers resistance to EPZ-5676.

To identify the EPZ-5676 resistance allele(s) produced by sgRNA #47, we performed deep sequencing analysis of the exon 8 region of DOT1L KMT domain targeted by this sgRNA in the resistant cell populations (Fig 1D and 1G). In each of the independently generated CRISPR-induced resistant cell populations, we observed a pronounced abundance of a combination deletion/missense mutation in-frame allele that deleted two valines and substituted two flanking glutamic acid and lysine residues with methionines via point mutations (VVEL293MM) (Fig 1H and S1 Table). This allele was present in low abundance at the initial time point following transduction with the sgRNA library but came to dominate the cell population following continuous exposure to EPZ-5676 (S1 Table). The mutation site closely coincided with the predicted DSB site induced by sgRNA #47 and eliminated the sgRNA recognition sequence, consistent with an on-target effect (Fig 1H). Importantly, the VVEL293MM allele was not detected in the parental RN2c cells, and hence can be confidently attributed to CRISPR-mediated mutagenesis. To further evaluate whether the VVEL293MM allele conferred resistance to EPZ-5676, we retrovirally expressed wild-type or VVEL293MM DOT1L human cDNA in RN2c cells and performed a titration of EPZ-5676 and measured growth rates. Compared to wild type DOT1L, the VVEL293MM DOT1L-expressing cells were less sensitive to EPZ-5676 treatment (Fig 1I). Collectively, these findings suggest that DOT1L KMT domain-focused indel mutagenesis had produced a novel allele that renders leukemia cells resistant to EPZ-5676-mediated growth arrest.

### Biochemical analysis of DOT1L VVEL293MM lysine methyltransferase activity

**Fig 2:**
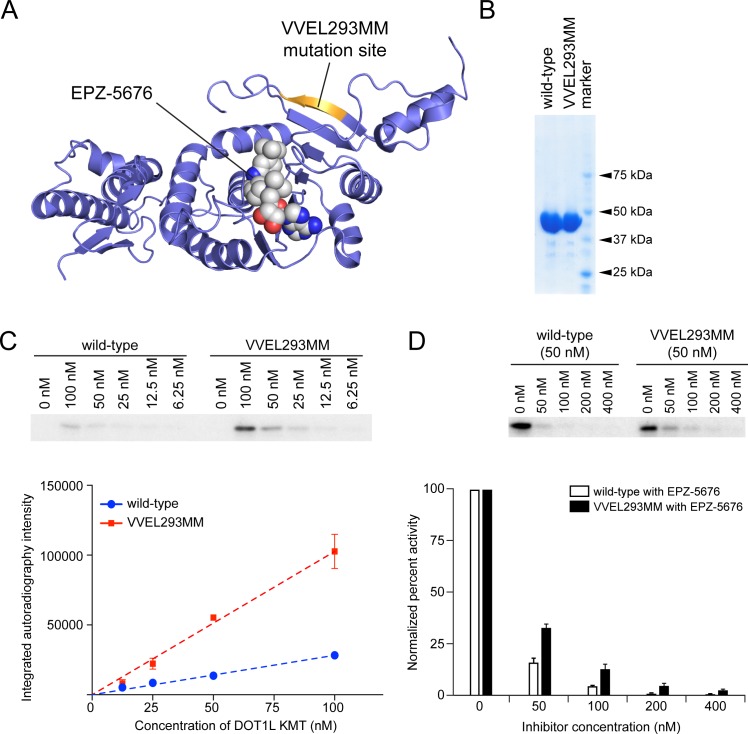
Biochemical analysis of DOT1L VVEL293MM lysine methyltransferase activity. (**A**) Crystal structure of DOT1L KMT domain binding with the EPZ-5676. The VVEL293MM mutation site was colored in yellow (PDB: 4HRA). (**B**) SDS-PAGE gel of purified recombinant DOT1L KMT domains of both wild-type and VVEL293MM mutant proteins. (**C**) Relative *in vitro* KMT activity of DOT1L wild-type and VVEL293MM mutant protein. The *in vitro* KMT activity was determined by a biochemical methyltransferase assay, which measured the methyltransfer level of radiolabeled SAM to purified nucleosomal substrates in the presence of a wild-type or mutant enzyme. To ensure that quantifications were made in the linear range of detection, the assay was performed at several DOT1L concentrations. The top panel shows a representative SDS-PAGE gel autoradiograph of the *in vitro* KMT assay with KMT proteins and concentrations of EPZ-5676 indicated. The lower panel plots the average integrated autoradiography intensity from two independent preparations of wild-type and mutant DOT1L KMT proteins. (**D**) Relative sensitivities of wild-type and VVEL293MM DOT1L to EPZ-5676. To normalize for the difference in basal activity between the wild-type and VVEL293MM DOT1L, the *in vitro* KMT assay reaction volume of the wild-type protein was increased three-fold. The top panel shows a representative SDS-PAGE gel autoradiograph of the *in vitro* KMT assay with KMT proteins and EPZ-5676 concentrations indicated. The lower panel plots the average results from three independent enzymatic preparations. All error bars shown represent s.e.m.

We next sought to understand the resistance mechanism of DOT1L VVEL293MM biochemically. Inspection of the position of the mutation on the available structure of the DOT1L KMT domain revealed that these residues were not directly involved in binding to EPZ-5676 [14] (Fig 2A), thus raising the possibility that the this mutation was conferring resistance to EPZ-5676 through an alternative mechanism, such as an enhancement of enzymatic activity. We performed a series of biochemical methyltransferase assays to evaluate this possibility using recombinant wild-type and VVEL293MM DOT1L KMT domains (Fig 2B), nucleosomal substrates, and radiolabeled S-adenosylmethionine (SAM). Remarkably, we observed that the VVEL293MM mutant was ~3.6 fold more active KMT relative to the wild-type enzyme (Fig 2C). Mass spectrometry analysis of these methylation reactions validated that H3K79 methylation was the predominant product of both wild-type and mutant DOT1L enzymes (S2 Fig). This finding was further corroborated by pre-methylating the substrates with non-radiolabeled SAM and wild-type DOT1L, which was found to block the ability of DOT1L VVEL293MM to methylate these nucleosomes (S2 Fig). Taken together, these findings demonstrate that DOT1L VVEL293MM allele has a hypermorphic, and not a neomorphic, function of H3K79 methyltransferase activity.

We next evaluated the relative sensitivities of the wild-type and VVEL293MM DOT1L to the EPZ-5676 inhibitor. We increased the reaction volume of the wild type protein by roughly three-fold in the *in vitro* methyltransferase activity assays to account for the differential basal level enzymatic activity between the wild-type and mutant domains. We observed that the VVEL293MM mutant enzyme was ~2-fold less sensitive to EPZ-5676 compared to the wild-type (Fig 2D). Collectively, these findings suggest that the VVEL293MM mutation confers two distinct effects on DOT1L KMT activity: it is a more active enzyme and it is marginally less sensitive to EPZ-5676. The combination of these two effects is likely to render leukemia cell growth less-sensitive to EPZ-5676, an effect that is analogous to the hypermorphic BRAF mutants conferred resistance to targeted therapy in melanoma [4].

### Domain-focused CRISPR genetic screening identifies an EZH2 mutant allele that is resistant to the drug EPZ-6438

To establish the robustness and adaptability of our method, we applied it to another epigenetic drug target. For this purpose, we focused on EZH2, a histone H3K27 methyltransferase and a catalytic subunit of the Polycomb Repressive Complex 2 (PRC2) [23]. EZH2 has been the focus of intense drug discovery efforts in recent years, and several compounds targeting this KMT have been described, including EPZ-6438 [15]. Moreover, prior studies have demonstrated that MLL-fusion leukemia cells are addicted to EZH2 enzymatic activity, and hence RN2c cell line is a suitable model for identifying resistance alleles [24–26].

**Fig 3:**
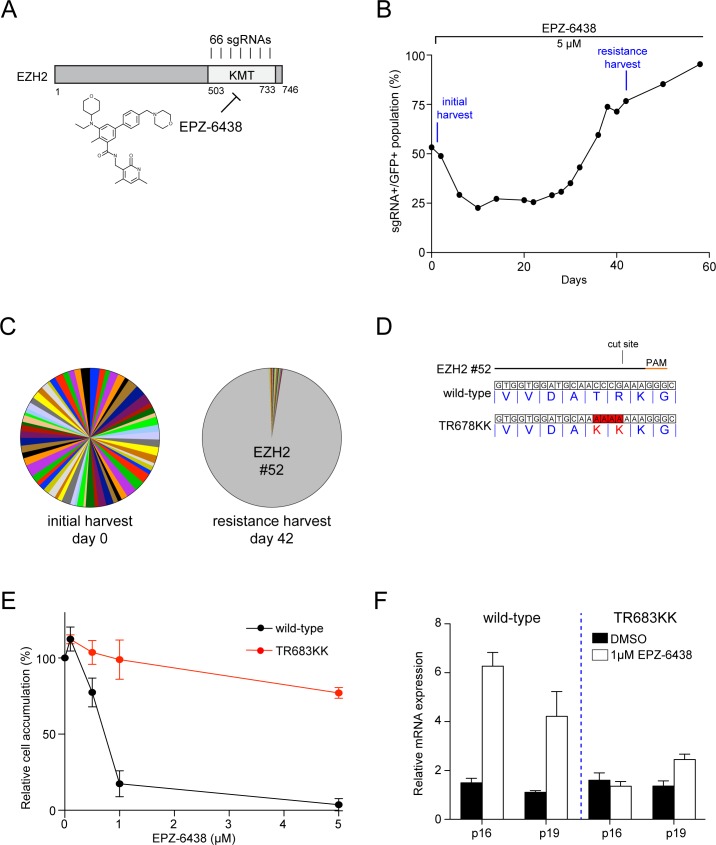
Domain-focused CRISPR genetic screening identifies an EZH2 mutant allele that is resistant to the drug EPZ-6438. (**A**) Schematic of EZH2 protein domain. A library of 66 sgRNAs was designed targeting the KMT domain of EZH2. The EZH2 inhibitor EPZ-6438 inhibits the KMT function. (**B**) CRISPR indel mutagenesis genetic screening identified EZH2 mutants resistant to EPZ-6438 treatment. RN2c cells were treated with 5 uM EPZ-6438 at day 3 post-transduction, at which point a fraction of the cells was harvested and used as a reference time point (day 0). At day 42, an EPZ-6438 resistant sgRNA+/GFP+ population was identified, harvested, and saved for subsequent deep sequencing experiments. (**C**) Pie charts of the relative abundance for individual 66 sgRNAs at indicated time points. Each slice represents the abundance of a single sgRNA. At day 0 (left), all 66 sgRNAs targeting the EZH2 KMT domain were detected at roughly equal abundance. At day 42 (right), EZH2 sgRNA #52 dominated the population, representing >95% of the GFP+ cells. (**D**) Nucleotide and protein sequence diagram of the wild-type and EZH2 mutation induced by sgRNA #52 CRISPR mutagenesis under the selection pressure of EPZ-6438. The indel mutations were observed in the exon 17 of EZH2 flanking the sgRNA cut site. Indel mutations were observed in exon 17 of EZH2 flanking the sgRNA cut site. The indel mutation corresponded to a TR to KK mutation from residue 678. (**E**) The effect of ectopic overexpression of EZH2 wild-type or TR683KK mutant on RN2c cell proliferation under the treatment of EPZ-6438. The RN2c cells were retrovirally transduced with wild-type or mutant human EZH2 cDNA. After a 5-day drug treatment, the relative cell accumulation corresponding to each indicated EPZ-6438 dosage was measured and normalized to the DMSO treated cells (*n* = 3). (**F**) qRT–PCR of relative mRNA expression levels in the indicated cell lines after a 5-day treatment with EPZ-5676 or DMSO. Results were normalized to *Gapdh*, with the relative mRNA level in the DMSO-treated cells set to 1 (*n* = 3). All error bars shown represent s.e.m.

To generate an EZH2 mutant that confers resistance to EPZ-6438 in cells and thus allows evaluation of on-target drug effects, we began with a domain-focused CRISPR indel mutagenesis screen analogous to the DOT1L screen described above, using a library of all possible sgRNAs (66 in total) targeting the KMT domain (Fig 3A). RN2c cells were then transduced with this library and cultured in the presence of 5 μM EPZ-6438. Similar to the observations with DOT1L, we initially observed a decline of sgRNA+/GFP+ cells (an indicator of EZH2 loss-of-function alleles being produced), followed by a gradual recovery of sgRNA+/GFP+ population after 20 days (Fig 3B). Importantly, the growth rate of RN2c cells at day 42 was dramatically less sensitive to EPZ-6438. A deep sequencing analysis of sgRNA abundance at early and late time-points revealed an over-representation of sgRNA #52 in the EPZ-6438-resistant cell population (Fig 3B). Moreover, Sanger sequencing of the EZH2 exonic target site of sgRNA #52 revealed an in-frame substitution allele, TR678KK, as the candidate resistance mutation (Fig 3D). To further establish causality between the TR678KK mutation and EPZ-6438 resistance, we cloned wild-type and mutant human EZH2 cDNA into a retroviral expression vector and tested whether overexpression of the mutation in RN2c cells could shift the sensitivity to EPZ-6438. Compared to wild-type, TR683KK EHZ2 cDNA overexpression rendered RN2c growth almost entirely resistant to EPZ-5676 (Fig 3E). Additionally, overexpression of TR683KK EZH2 cDNA also prevented the transcriptional up-regulation of PRC2-repressed genes following EPZ-6436 exposure (Fig 3F). These findings suggest that domain-focused CRISPR indel mutagenesis can be applied more broadly to epigenetic targets to generate novel resistance alleles, which can be used as definitive tools for evaluating on-target effects of suitable chemical probes.

## Discussion

In this study we describe a method that employs CRISPR-Cas9 indel mutagenesis to rapidly derive alleles of endogenous KMT genes that allow definitive demonstration of on-target effects of chemical inhibitors. While the overwhelming majority of CRISPR-induced indel mutations are likely to be loss-of-function, particularly when targeting critical enzymatic domains [11,13], we have shown that rare in-frame alleles can be produced that retain functionality, while simultaneously preventing direct-acting small-molecule inhibitors from causing cellular growth phenotypes. DOT1L provides an interesting example, in which the allele that renders cell growth less sensitive to a DOT1L inhibitor works mainly through enhancement of KMT activity. This example is analogous to observations in BRAF, EGFR, and HER2, in which elevated kinase activity allowed cells to grow in the presence of kinase inhibitors [4,27]. In the case of DOT1L VVEL293MM, it is likely that enhanced basal level of H3K79 methylation of endogenous chromatin produced by this allele requires a much higher dose of EPZ-5676 to sufficiently decrease H3K79 methylation levels below the critical threshold to suppress HOX and MEIS1 expression thereby limiting leukemia growth. However, it is likely that the modest 2-fold decrease in inhibitor sensitivity of VVEL293MM also contributes to the resistance phenotype as well. In future work, it will be interesting to evaluate whether indel-mediated resistance mechanisms generated via CRISPR will favor hypermorphic enzyme mechanisms instead of abrogation of direct chemical inhibitor binding. During the preparation of this manuscript, a study by Donovan et al reported a similar conclusion as ours, by showing that CRISPR-Cas9 indel mutagenesis can generate gain-of-function or inhibitor-resistant alleles of several kinase targets [28]. Taken together, our two studies suggest that CRISPR-based indel mutagenesis can be applied to diverse target classes to validate on-target activity of inhibitors in cells.

The approach described here is complementary to other recently described strategies for generating resistance alleles. We recently reported the use of domain-swapping as a means to generate resistance alleles, using the bromodomain protein BRD9 and EZH2 as targets [16]. Recently, several groups have reported methods to create mutants in an endogenous genetic locus by tethering catalytically dead Cas9 protein with a cytidine deaminase (CRISPR-AID) to induce base pair mismatches [29,30]. Through manipulation of the DNA mismatch repair pathway, CRISPR-AID could also generate a pool of missense mutations for screening chemical inhibitor-resistant mutants [31,32]. The indel mutations generated through error-prone repair pathways provide a different mutational spectrum that is complementary to the missense mutations induced by the CRISPR-AID strategies. In this regard, we expect that the combination of CRISPR-mutagenesis and CRISPR-AID methods to be a powerful tool for generating inhibitor-resistant mutants and gain-of-function proteins.

## Supporting information

S1 Fig A proliferation competition assay of RN2c cells transduced with indicated sgRNAs under the treatment of EPZ-5676 or DMSO shows that DOT1L sgRNA #47 induced mutant(s) confer RN2c resistance to EPZ-5676. An sgRNA targeting a Rosa locus and a random sgRNA targeting DOT1L KMT domain (DOT1L sgRNA #64) served as negative controls.
(PDF)

S2 Fig DOT1L VVEL293MM does not exhibit neomorphic activity on nucleosome substrates. (**A**) iTRAQ experiments did not detect neomorphic methytransferase activity for the mutant enzyme. All methylated peptides from the experiment are tabulated and shown as raw iTRAQ counts (top) or iTRAQ ratios normalized to the wild-type protein reaction (bottom). (**B**) Nucleosomes that were first incubated with wild-type enzyme and S-adenosyl methionine did not show subsequent transfer of radiolabled methyl groups in the presence of either additional wild-type (lane 1) or mutant protein (lane 2). Both preparations of the enzyme were active as demonstrated by radiolabeled methyl transfer (lanes 3 and 4), consistent with previous observations.
(PDF)

S1 Table DOT1L KMT domain pooled screen data and DNA oligo sequences. (XLSX)

## References

[1] Azam M, Latek RR, Daley GQ (2003) Mechanisms of autoinhibition and STI-571/imatinib resistance revealed by mutagenesis of BCR-ABL. Cell 112: 831–843. 12654249 10.1016/s0092-8674(03)00190-9

[2] Botstein D, Shortle D (1985) Strategies and applications of in vitro mutagenesis. Science 229: 1193–1201. 2994214 10.1126/science.2994214

[3] Poulikakos PI, Persaud Y, Janakiraman M, Kong X, Ng C, et al (2011) RAF inhibitor resistance is mediated by dimerization of aberrantly spliced BRAF(V600E). Nature 480: 387–390. 10.1038/nature10662 22113612 PMC3266695

[4] Foster SA, Whalen DM, Ozen A, Wongchenko MJ, Yin J, et al (2016) Activation Mechanism of Oncogenic Deletion Mutations in BRAF, EGFR, and HER2. Cancer Cell 29: 477–493. 10.1016/j.ccell.2016.02.010 26996308

[5] Hsu PD, Lander ES, Zhang F (2014) Development and applications of CRISPR-Cas9 for genome engineering. Cell 157: 1262–1278. 10.1016/j.cell.2014.05.010 24906146 PMC4343198

[6] Doudna JA, Charpentier E (2014) Genome editing. The new frontier of genome engineering with CRISPR-Cas9. Science 346: 1258096 10.1126/science.1258096 25430774

[7] Cong L, Ran FA, Cox D, Lin S, Barretto R, et al (2013) Multiplex genome engineering using CRISPR/Cas systems. Science 339: 819–823. 10.1126/science.1231143 23287718 PMC3795411

[8] Jinek M, Chylinski K, Fonfara I, Hauer M, Doudna JA, et al (2012) A programmable dual-RNA-guided DNA endonuclease in adaptive bacterial immunity. Science 337: 816–821. 10.1126/science.1225829 22745249 PMC6286148

[9] Mali P, Yang L, Esvelt KM, Aach J, Guell M, et al (2013) RNA-guided human genome engineering via Cas9. Science 339: 823–826. 10.1126/science.1232033 23287722 PMC3712628

[10] Jasin M, Haber JE (2016) The democratization of gene editing: Insights from site-specific cleavage and double-strand break repair. DNA Repair (Amst). 10.1016/j.dnarep.2016.05.001 PMC5529214 27261202

[11] Shi J, Wang E, Milazzo JP, Wang Z, Kinney JB, et al (2015) Discovery of cancer drug targets by CRISPR-Cas9 screening of protein domains. Nat Biotechnol 33: 661–667. 10.1038/nbt.3235 25961408 PMC4529991

[12] Moreno-Mateos MA, Vejnar CE, Beaudoin JD, Fernandez JP, Mis EK, et al (2015) CRISPRscan: designing highly efficient sgRNAs for CRISPR-Cas9 targeting in vivo. Nat Methods 12: 982–988. 10.1038/nmeth.3543 26322839 PMC4589495

[13] Munoz DM, Cassiani PJ, Li L, Billy E, Korn JM, et al (2016) CRISPR Screens Provide a Comprehensive Assessment of Cancer Vulnerabilities but Generate False-Positive Hits for Highly Amplified Genomic Regions. Cancer Discov. 10.1158/2159-8290.CD-16-0178 27260157

[14] Daigle SR, Olhava EJ, Therkelsen CA, Basavapathruni A, Jin L, et al (2013) Potent inhibition of DOT1L as treatment of MLL-fusion leukemia. Blood 122: 1017–1025. 10.1182/blood-2013-04-497644 23801631 PMC3739029

[15] Knutson SK, Warholic NM, Wigle TJ, Klaus CR, Allain CJ, et al (2013) Durable tumor regression in genetically altered malignant rhabdoid tumors by inhibition of methyltransferase EZH2. Proc Natl Acad Sci U S A 110: 7922–7927. 10.1073/pnas.1303800110 23620515 PMC3651445

[16] Hohmann AF, Martin LJ, Minder JL, Roe JS, Shi J, et al (2016) Sensitivity and engineered resistance of myeloid leukemia cells to BRD9 inhibition. Nat Chem Biol. 10.1038/nchembio.2115 PMC4990482 27376689

[17] Zuber J, McJunkin K, Fellmann C, Dow LE, Taylor MJ, et al (2011) Toolkit for evaluating genes required for proliferation and survival using tetracycline-regulated RNAi. Nat Biotechnol 29: 79–83. 10.1038/nbt.1720 21131983 PMC3394154

[18] Morita S, Kojima T, Kitamura T (2000) Plat-E: an efficient and stable system for transient packaging of retroviruses. Gene Ther 7: 1063–1066. 10.1038/sj.gt.3301206 10871756

[19] Nguyen AT, Zhang Y (2011) The diverse functions of Dot1 and H3K79 methylation. Genes Dev 25: 1345–1358. 10.1101/gad.2057811 21724828 PMC3134078

[20] Bernt KM, Zhu N, Sinha AU, Vempati S, Faber J, et al (2011) MLL-rearranged leukemia is dependent on aberrant H3K79 methylation by DOT1L. Cancer Cell 20: 66–78. 10.1016/j.ccr.2011.06.010 21741597 PMC3329803

[21] Daigle SR, Olhava EJ, Therkelsen CA, Majer CR, Sneeringer CJ, et al (2011) Selective killing of mixed lineage leukemia cells by a potent small-molecule DOT1L inhibitor. Cancer Cell 20: 53–65. 10.1016/j.ccr.2011.06.009 21741596 PMC4046888

[22] van Overbeek M, Capurso D, Carter MM, Thompson MS, Frias E, et al (2016) DNA Repair Profiling Reveals Nonrandom Outcomes at Cas9-Mediated Breaks. Mol Cell 63: 633–646. 10.1016/j.molcel.2016.06.037 27499295

[23] Margueron R, Reinberg D (2011) The Polycomb complex PRC2 and its mark in life. Nature 469: 343–349. 10.1038/nature09784 21248841 PMC3760771

[24] Xu B, On DM, Ma A, Parton T, Konze KD, et al (2015) Selective inhibition of EZH2 and EZH1 enzymatic activity by a small molecule suppresses MLL-rearranged leukemia. Blood 125: 346–357. 10.1182/blood-2014-06-581082 25395428 PMC4287641

[25] Shi J, Wang E, Zuber J, Rappaport A, Taylor M, et al (2013) The Polycomb complex PRC2 supports aberrant self-renewal in a mouse model of MLL-AF9;Nras(G12D) acute myeloid leukemia. Oncogene 32: 930–938. 10.1038/onc.2012.110 22469984 PMC4102143

[26] Neff T, Sinha AU, Kluk MJ, Zhu N, Khattab MH, et al (2012) Polycomb repressive complex 2 is required for MLL-AF9 leukemia. Proc Natl Acad Sci U S A 109: 5028–5033. 10.1073/pnas.1202258109 22396593 PMC3324004

[27] Chen SH, Zhang Y, Van Horn RD, Yin T, Buchanan S, et al (2016) Oncogenic BRAF Deletions That Function as Homodimers and Are Sensitive to Inhibition by RAF Dimer Inhibitor LY3009120. Cancer Discov 6: 300–315. 10.1158/2159-8290.CD-15-0896 26732095

[28] Donovan KF, Hegde M, Sullender M, Vaimberg EW, Johannessen CM, et al (2017) Creation of Novel Protein Variants with CRISPR/Cas9-Mediated Mutagenesis: Turning a Screening By-Product into a Discovery Tool. PLoS One 12: e0170445 10.1371/journal.pone.0170445 28118392 PMC5261743

[29] Komor AC, Kim YB, Packer MS, Zuris JA, Liu DR (2016) Programmable editing of a target base in genomic DNA without double-stranded DNA cleavage. Nature 533: 420–424. 10.1038/nature17946 27096365 PMC4873371

[30] Nishida K, Arazoe T, Yachie N, Banno S, Kakimoto M, et al (2016) Targeted nucleotide editing using hybrid prokaryotic and vertebrate adaptive immune systems. Science 353. 10.1126/science.aaf8729 27492474

[31] Hess GT, Fresard L, Han K, Lee CH, Li A, et al (2016) Directed evolution using dCas9-targeted somatic hypermutation in mammalian cells. Nat Methods 13: 1036–1042. 10.1038/nmeth.4038 27798611 PMC5557288

[32] Ma Y, Zhang J, Yin W, Zhang Z, Song Y, et al (2016) Targeted AID-mediated mutagenesis (TAM) enables efficient genomic diversification in mammalian cells. Nat Methods 13: 1029–1035. 10.1038/nmeth.4027 27723754

