# Supplementary material for: Rapid generation of drug-resistance alleles at endogenous loci using CRISPR-Cas9 indel mutagenesis
Source: PLoS One. 2017 Feb 23;12(2):e0172177. doi: 10.1371/journal.pone.0172177 (PMC5322889; doi:10.1371/journal.pone.0172177)
Supplement: S1 Fig — An sgRNA targeting a Rosa locus and a random sgRNA targeting DOT1L KMT domain (DOT1L sgRNA #64) served as negative controls. (PDF) [file pone.0172177.s001.pdf]

# Supporting information

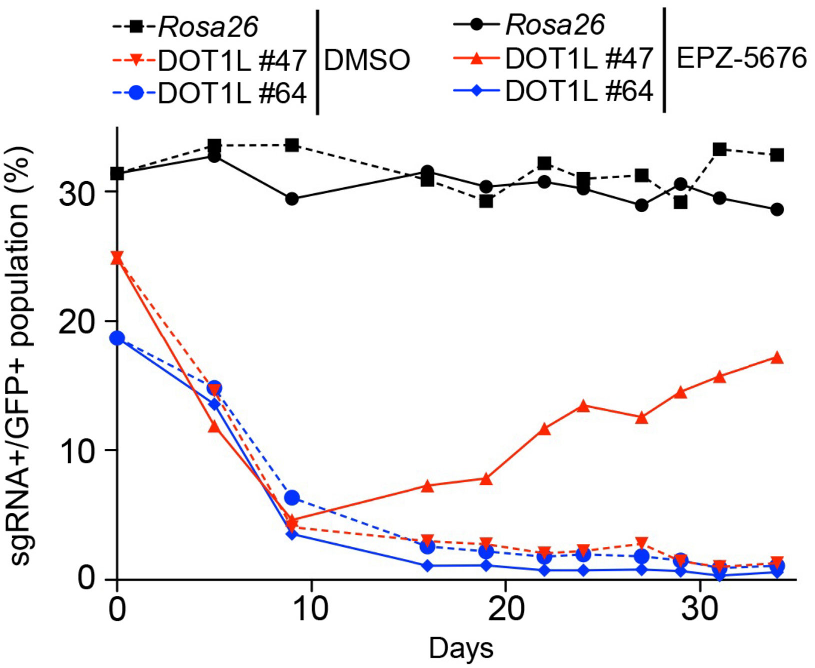


**S1 Fig.** A proliferation competition assay of RN2c cells transduced with indicated sgRNAs under the treatment of EPZ-5676 or DMSO show that DOT1L sgRNA #47 induced mutant(s) confer RN2c resistance to EPZ-5676. An sgRNA targeting a Rosa locus and a random sgRNA targeting DOT1L KMT domain (DOT1L sgRNA #64) served as negative controls.
