# Supplementary material for: Rapid generation of drug-resistance alleles at endogenous loci using CRISPR-Cas9 indel mutagenesis
Source: PLoS One. 2017 Feb 23;12(2):e0172177. doi: 10.1371/journal.pone.0172177 (PMC5322889; doi:10.1371/journal.pone.0172177)
Supplement: S2 Fig — (A) iTRAQ experiments did not detect neomorphic methytransferase activity for the mutant enzyme. All methylated peptides from the experiment are tabulated and shown as raw iTRAQ counts (top) or iTRAQ ratios normalized to the wild-type protein reaction (bottom). (B) Nucleosomes that were first incubated with wild-type enzyme and S-adenosyl methionine did not show subsequent transfer of radiolabled methyl groups in the presence of either additional wild-type (lane 1) or mutant protein (lane 2). Both preparations of the enzyme were active as demonstrated by radiolabeled methyl transfer (lanes 3 and 4), consistent with previous observations. (PDF) [file pone.0172177.s002.pdf]

A

Raw iTRAQ counts

protein description	peptide sequence	peptide modification(s)	wildtype	VVEL293MM	nucleosomes	nucleosomes
Histon H3.1 (human)	DIQLAR		2348	2349.9	2834	2865
Histon H3.1 (human)	EIAQDFK iTRAQ	8plex (K)	641.42	177.85	4692.1	6090.3
Histon H3.1 (human)	EIAQDFKTDLR	Dimethyl (K)	1689	1871	92.33	0
Histon H3 (human)	FQSAAIGALQEASEAYLVGLFEDTNLCAIHAK	iTRAQ8plex (K)	97.44	139.66	100.5	82.32
Histon H3.1 (human)	FQSSAVMALQEACEAYLVGLFEDTNLCAIHAK	iTRAQ8plex (K)	50.37	201.61	183.53	143.49
Histon H3.2 (human)	FQSSAVMALQEASEAYLVGLFEDTNLCAIHAK	iTRAQ8plex (K)	212.85	217.31	37.39	43.53

*Other methylation detected in all proteins			wildtype	VVEL293MM	nucleosomes	nucleosomes
Heat shock cognate 71 kDa	IINEPTAAAIAYGLDK	Dimethyl (K)	349.5	124.7	90.02	119.2
Dot1L (human)	SPVGAEPAVYPWPLPVYDKHHDAAHEIIETIR	Trimethyl (K)	26.76	27.63	0	0

iTRAQ ratios

protein description	peptide sequence	peptide modification(s)	wildtype	VVEL293MM	nucleosomes	nucleosomes
Histon H3.1 (human)	DIQLAR		1	1.00	1.21	1.22
Histon H3.1 (human)	EIAQDFK	iTRAQ 8plex (K)	1	0.28	7.32	9.50
Histon H3.1 (human)	EIAQDFKTDLR	Dimethyl (K)	1	1.11	0.05	0
Histon H3 (human)	FQSAAIGALQEASEAYLVGLFEDTNLCAIHAK	iTRAQ8plex (K)	1	1.43	1.03	0.84
Histon H3.1 (human)	FQSSAVMALQEACEAYLVGLFEDTNLCAIHAK	iTRAQ8plex (K)	1	4.00	3.64	2.85
Histon H3.2 (human)	FQSSAVMALQEASEAYLVGLFEDTNLCAIHAK	iTRAQ8plex (K)	1	1.02	0.18	0.20

*Other methylation detected in all proteins			wildtype	VVEL293MM	nucleosomes	nucleosomes
Heat shock cognate 71 kDa	IINEPTAAAIAYGLDK	Dimethyl (K)	1	0.357	0.258	0.341
Dot1L (human)	SPVGAEPAVYPWPLPVYDKHHDAAHEIIETIR	Trimethyl (K)	1	1.033	0	0

B

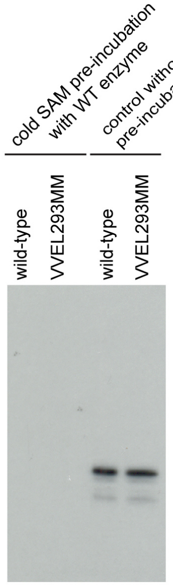

S2 Fig. DOT1L VVEL293MM does not exhibit neomorphic activity on nucleosome substrates. (**A**) iTRAQ experiments did not detect neomorphic methytransferase activity for the mutant enzyme. All methylated peptides from the experiment are tabulated and shown as raw iTRAQ counts (top) or iTRAQ ratios normalized to the wild-type protein reaction (bottom). (**B**) Nucleosomes that were first incubated with wild-type enzyme and S-adenosyl methionine did not show subsequent transfer of radiolabled methyl groups in the presence of either additional wild-type (lane 1) or mutant protein (lane 2). Both preparations of the enzyme were active as demonstrated by radiolabeled methyl transfer (lanes 3 and 4), consistent with previous observations.
